# Supplementary material for: In-stream habitat availability for river dolphins in response to flow: Use of ecological integrity to manage river flows
Source: PLoS One. 2021 Jul 21;16(7):e0241099. doi: 10.1371/journal.pone.0241099 (PMC8294540; doi:10.1371/journal.pone.0241099)
Supplement: Attachment — Submitted filename: Response _to reviewers.docx [file pone.0241099.s002.docx]

Date: 12-29-2020

Susana Caballero, PhD

Academic editor
PLOS ONE

Dear Susana,

First of all, I would like to thank you for the opportunity to revise and resubmit our manuscript, entitled “***In-stream habitat availability for river dolphins in response to flow: use of ecological integrity to manage river flows***”. I found the reviewers’ comments to be helpful in revising the manuscript and have carefully considered and responded to each suggestion. **We completely addressed issues raised by the reviewer two but in the case of reviewer one, the reviewer seems like deviated from the core aim and approach of the paper due to technical limitations that connect ecology-flow. Since this paper is extended findings of the research which was recently published in Nature Scientific Reports (**[Ecological responses to flow variation inform river dolphin conservation | Scientific Reports (nature.com)](https://www.nature.com/articles/s41598-020-79532-3)) **that builds flow-ecology relationships**, **the approach mentioned in the submitted paper to PLOS ONE is scientifically and technically validated (the same approach we published in Nature Scientific Reports was used). The reviewer mentioned technically inadequate, and recommend to consult the hydrologist. However, we had a team of biologists, hydrologist, geologist and engineers, who made this approach collectively to develop the flow-ecology relationships and now scientifically accepted as indicated by the publication in Nature Scientific Reports. Even though we mentioned clear measurement approach (available and occupied hydro-physical habitats) and links between flow-ecology through AWS calculation, the reviewer one finds hard to connect the links between flow-ecology. We added lines (199-203) and references [10, 33; above published paper] to make the approach and goal clearer to the reader. This is clearly mentioned in lines 118- 126 and 169-206. Again, the approach mentioned in this paper is already published that indicates technically and scientifically validated.**

I have included a response to reviewers (below) in which we address each comment the reviewers made. In our response to reviewers, our responses follow immediately below the comment in yellow highlight. Corresponding changes are highlighted in yellow in the manuscript text in the revised file.

Important notes:

1. **Please include this funding statement in the system on behave of author: *"The author (SP) received field research grants from: Rufford Foundation (UK), Commonwealth Scientific and Industrial Research Organization (CSIRO-Australia), and WWF-Nepal. The funders had no role in study design, data collection and analysis, decision to publish, or preparation of the manuscript."***
2. **Figure 1 is already published [ reference 10] written by same principal author (Shambhu Paudel). This figure is re-use under the CC BY License agreement.**

Thank you again for your consideration of our revised manuscript.

Sincerely,

Shambhu Paudel, Ph.D. scholar

School of Natural Resource and the Environment

University of Arizona, Tucson, Arizona, USA

And

Institute of Forestry, Pokhara, Nepal

Email: [spaudel@email.arizona.edu](mailto:spaudel@email.arizona.edu)

**Response to reviewers:**

Editor Comments:

I received two reviews for this manuscript. Both reviewers commend the authors on the work and they think this type of research is needed urgently to further understand ways in which river dolphins can be conserved and viable populations can be maintained. However, one of the reviewers, an expert in hydraulics, was critic considering the way in which the authors explain the concepts related to hydraulics. I cite him: "noted a few occasions when the authors did not appear to clearly understand what they were describing, and overall I felt that the presentation quality of the hydraulic results was low. If possible I would suggest collaborating with someone in that field as the data set looks promising and the problem clearly merits attention. I have made many other comments in the manuscript".

As this study combines river flow property and the ecology of the river dolphins to predict the in-stream habitat availability of river dolphins, the first reviewer finds it hard to understand its core approach and idea. Principally, we are not entirely working on modeling river hydraulics as does a hydrologist, but we quantify in-stream area availability to river dolphins as a function of river depth and flow at a particular flow level. To quantify habitat availability, we use ADP, which measures the complete cross-sectional dynamics using acoustics and provide us data on depth and velocity at cell size between 0.02-0.5 m, including cross-sectional area, width, discharge. Using hydro-physical properties of habitats with documented river dolphin use, we develop a habitat suitability curve, and then it was forwarded to estimate area-weighted suitability (AWS) measured in m^2^/m. AWS is the available suitable habitats at each cross-section. This details of the entire approach are described in lines 118-126 (introduction) and 169-206 (methods)

We also added a simple overview to the approach by adding lines 199-203 and a new reference in line 186.

The second reviewer, an expert in river dolphin conservation was very positive about the manuscript and considered acceptance.

The second reviewer who is river cetacean scientist did completely understand the approach that combines ecology and flow. We believe our additional details and a short overview should satisfy the breadth of readership that we suspect will find the paper useful. We appreciate the comments of Rev 1 and you to create a more accessible manuscript. Thank you!

Considering these two reviews, I suggest the authors that they thoroughly revise their manuscript, especially the concepts and results related with hydraulics. Maybe communicate with a local expert in this field that can provide their expertise?

Our paper that details some of the methods was just published and we now cite that reference here in this PLoS ONE paper and that should further help:

[Ecological responses to flow variation inform river dolphin conservation | Scientific Reports (nature.com)](https://www.nature.com/articles/s41598-020-79532-3)

We had a team of geologists, hydrologists and biologist, who designed the research approach, estimated the AWS and were instrumental in the general approach detailed in the Nature Sci Reports paper and applied in our manuscript submitted manuscript to PLoS ONE. We believe that our added details and the reference to the recent publication will assist with the accessibility of this work and hope that you agree!

I personally consider that we need all the good quality information that can be made available for river dolphin conservation, so I really hope the authors work on this recommendation to improve the quality of their paper.

As we already published the first outcome of this project, this paper, with the same approach, we elaborated further details about the river dolphin’s habitat in relation to the flow. The approach adopted by this paper was the same that we used in the research paper published in Nature Scientific reports ([Ecological responses to flow variation inform river dolphin conservation | Scientific Reports (nature.com)](https://www.nature.com/articles/s41598-020-79532-3)). I hope this helps with the decision and ensures that we used a technically sound approach to make this paper scientifically robust.

Reviewer #1: I read this study with interest as it did describe a novel approach to understanding the habitat of river dolphins, which is critical, as the authors say, for understanding what the impacts of flow regulation in the form of hydropower megaprojects will be on habitat loss and fragmentation. As a researcher on the hydraulic side, this seemed to me an important opportunity to delve into an important issue of our time and to link it with channel hydraulics. I am not an expert on the AWS modelling and the statistical tools used to asses the model and so will not comment extensively on them.

The aim of this paper is to estimate AWS as a function of velocity and depth at the finer spatial resolution at a particular flow level. We estimated the AWS as each cross-section in response to flow level. It is the primary or core goal of this paper to inform practice and policy. We have added more details and an overview of the technique to assist with this understanding.

I commend the researchers on their efforts to obtain a unique and important dataset in this environment. However, the study should not be considered adequate from a hydraulic perspective.

We have changed some of our wording as mentioned above and provided more details as we are not particularly dealing with hydraulic modeling. Here, we combine flow (velocity and depth at particular flow level) with reference to dolphin requirements to develop flow-ecology relationships in terms of in-stream habitat availability (AWS). This is the goal of this paper … an application of the hydrological characteristics to the ecology and conservation of river dolphins. To complete this task, we measured a continuum available hydro-physical habitat (line 147) and occupied habitats by river dolphins (line 191) to develop the suitable habitat curve. This curve is further used to estimate AWS at each cross-section. Here we define river dolphin habitat by the interaction of velocity and depth (lines 118-120), which were measured by ADP at 0.02 to 0.5m spatial resolution across the cross-section. We tweaked the details of this approach in the methods section. This approach is the standard way to estimate habitat suitability; we have published the methods in greater detail elsewhere and now reference them as noted above.

I noted a few occasions when the authors did not appear to clearly understand what they were describing, and overall I felt that the presentation quality of the hydraulic results was low.

We added further lines (199-203) and two references [10, 33] to make approach clearer to the reader.

If possible I would suggest collaborating with someone in that field as the data set looks promising and the problem clearly merits attention.

This is a project with a team member representing diverse areas, from ecologist, biologist, geologist, hydrologist to engineers. We have published the details elsewhere as noted above but we add more detail and citation to assist with the reader with the approach.

I have made many other comments in the manuscript,

We accepted all the comments made in the manuscript, which are highlighted by yellow color.

but overall my recommendation is to reject.

I think we have made the approach clearer, added information and references to improve the quality of the paper.

Reviewer #2: The manuscript is well prepared and develops the objectives proposed in the investigation in a clear and robust way.

Thank you! Yes, we appreciate that you found the details clear and the manuscript valuable.

Please change the term "riverine cetaceans" in the document to "river dolphins", and in the abstract section in the lines 30 -31 change the words "with reference to" for concerning or regarding. Introduction in the line 59 delete the words "the", in the line 67 delete ",", line 94 include "," in the phrase "species, and", line 103 "the" in the phrase "the presence", and change the word "driven" for drove or has driven, line 107 include "," the word "however", line 108 include "the" in the phrase "the quality", line 129 change the phrase " in relation to" for "about, to, with or concerning", and change the phrase "properties of wider level" for "properties of a wider level". Materials and Methods in the line 141 change the words "endangered" for "endanger", line 177 change the words "cross section" for "cross-section", line 192 remove "," of the phrase "considered pools, and intermediate", line 201 change the word "main stream" or "mainstream", line 202 change the word "characteristic" for "characteristics", lines 231 and 238 change the word "dropterm" for "drop term", lines 241 and 247 include "the" in the phrase " in the GAMLSS package to", and "were completed using the gamlss package". Results in the line 252 include the word "the season" or "a season", line 254 include the word "The higher", and "the lowest", line 275 include "," in the phrase " , and", line 278 include "the coefficient", and line 295 change the word "were flowed or were flowing". Discussion in the line 363 include "-" in the phrase "broad-scale", line 364 include "a" in the phrase "a strong", line 382 include " the river", line 390 change the word "is" for "are", line 413 include " with an average", line 436 delete "a" in the phrase "with similar velocity", line 438 change the word "indicates" or "indicate", line 446 in include the word "that the vulnerability", line 447 change the word "is" for "are" and include the word "the flow", line 488 include the word "the development", line 449 include "the viability", and line 496 change the word "under" for "at".

We appreciate the suggestions and make all changes highlighted in yellow color in the manuscript and agree that the readability is improved.
