# Supplementary material for: In-stream habitat availability for river dolphins in response to flow: Use of ecological integrity to manage river flows
Source: PLoS One. 2021 Jul 21;16(7):e0241099. doi: 10.1371/journal.pone.0241099 (PMC8294540; doi:10.1371/journal.pone.0241099)
Supplement: Attachment — Submitted filename: Response to Reviwers.docx [file pone.0241099.s003.docx]

Date: 05-22-2021

Susana Caballero, PhD

Academic editor
PLOS ONE

Dear Susana,

First of all, I would like to thank you for the opportunity to revise and resubmit our manuscript, entitled “***In-stream habitat availability for river dolphins in response to flow: use of ecological integrity to manage river flows***”. I found the reviewers’ comments to be helpful in revising the manuscript and have carefully considered and responded to each suggestion. **We completely addressed issues raised by the reviewers.**

I have included a response to reviewers (below) in which we address each comment the reviewers made. In our response to reviewers, our responses follow immediately below the comment in yellow highlight. Corresponding changes are highlighted in yellow in the manuscript text in the revised file.

Thank you again for your consideration of our revised manuscript.

Sincerely,

Shambhu Paudel, Ph.D. scholar

School of Natural Resource and the Environment

University of Arizona, Tucson, Arizona, USA

And

Institute of Forestry, Pokhara, Nepal

Email: [spaudel@email.arizona.edu](mailto:spaudel@email.arizona.edu)

Reviwer#1:

1. At line 183, I’m still not sure where this definition of the Froude number comes from. You may be thinking of the Reynolds number, which is a better index of turbulence because it is the ratio of the inertial to the viscous force. Froude number is a ratio of the inertial to the gravitational force and is an index of wave behaviour. In rivers it is used to understand transitions between sub and supercritical flow. This poor definition of the Froude number was one of the main reasons I suggested working with someone with a better knowledge of open channel flow hydraulics.

Thank you for your suggestion. We follow Jowett (1993-A method for objectively identifying pool, run, and riffle habitats from physical measurements) and System for Environmental Flow Analysis (SEFA; Jowett et al. 2019) software manual to classify the habitats into the pool, run and riffle habitats from physical measurements taking the Froude number as an index of hydraulic turbulence. The reference papers compare the hydraulic characteristics of these three habitats and derive a simple habitat classification criterion which we applied in our study. This is a simple classification rule which correctly classified 66% of the habitats (Jowett, 1993). We have added some of these statements (including reference) to the methods section (lines 184-187) and then the reviewer for requesting more clarity.

2. At line 186 it is good to see that a reference is now provided to indicate the source for these Froude number thresholds. I note, however, that these numbers are from a single braided river in the Alps (Jowett, 1993). They are applied uncritically in this case to a much different river in terms of depth and velocity. I very much doubt that the morphologic description of riffles and pools from shallow braided river would apply to this much larger river where dolphins reside. The values used for the Froude number-based classification system is thus still not well justified.

As this is the first study in the region that develops flow-ecology relationships taking behavioral ecology of a large predator, the adopted approaches can be revised or revisited in the future study as this approach correctly classified 66% of the habitats. Previous studies visually classified the habitat types; however, we consider hydraulic properties measured by ADP at a higher spatial scale to classify the habitat. At the initial phase of this project, we consulted our approach with a senior hydrologists and key stakeholders in Nepal who are responsible for aquatic species and flow management before conducting field sampling. Thus, we have adopted the generally acceptable and simple approach to standardize the fieldwork using state-of-the-art technology so that these can be applied by biologists in the field. We appreciate the reviewer’s concern and have added a statement about the need for further testing and refinement of habitat classification in the future (lines: 189-194).

3. For the GAMLSS models and results presented in Table 3, the Habitat Type (HT) is based on Froude number, which is based on Velocity and Depth. What is the benefit or logic of including Velocity Depth and Habitat Type on the model? Can't you do it with Velocity and Depth alone? Based on Figure 3, is habitat type really useful? It still seems to me that you are double counting or mixing hydraulic variables. Riffles have high velocity, but low depth, which according to the other plots have opposite effects on AWS. Did you test any models without HT? In Table 3 they all have it.

First, we develop the full linear model using all the available variables to avoid bias on parameter selection. Then to get the final model with only significant terms, stepwise model selection step applied which helps to select the top model based on GAIC. This indicates parameters are included or excluded systematically based on significant contribution to the response variable (AWS).

After having these linear, interactive, and smoother terms which develop the full initial model, we again use a stepwise selection process based on GAIC. All models listed in the table are developed following the modeling procedure that considers only significant contributor and removes non-relevant elements by the stepwise process systematically.

We explicitly described all the statistical steps described above or that we followed in the lines 219-253.

4. Figure 7 is still poor quality. There no axes labels, labels on the color bars, or indications of what the contours mean and the contour labels are too small to read.

We included a high-quality image with label and color bar in the revised manuscript. We included a high-quality image with label and color bar in the revised manuscript. We submitted journal standard quality, however, while creating a PDF for review, it loses its quality. We uploaded high quality individual file of each maps separately in the system for the final publication.

5. In the response to my comments the authors state that they are ‘not particularly dealing with hydraulic modeling’, but they are using hydraulic measurements and concepts and using those to describe the habitat of these river dolphins. In offering my opinion I am not deviating from the core aim of your paper as you state in the letter, but simply trying to ensure that sound science is being published. I am judging the work solely based on what is in the current paper and have not consulted the Nature paper, which, while a significant accomplishment, is not the subject of the current review. The manuscript in front of me has some serious flaws related to the core idea that you are describing the hydraulic habitat of these creatures and these have not been addressed by your revision.

This paper aims to quantify in-stream habitat availability using the predominantly applied Habitat Suitability Curve (HSC) approach (see application and relevance of HSC in aquatic ecology- Nestler et al. 2019). This approach is one of several approaches used in sustainable flow management while balancing societal demands and conservation. We built the HSC (based on velocity and depth) to estimate AWS (area-weighted suitability), the steps to develop HSC are explicitly described in Paudel et al. 2020 and cited in this paper as well to facilitate the learning process for the audience. Only the interactive function of velocity and depth was used to estimate AWS. We applied a widely consider approach to quantify the in-stream habitat availability in response to flow, but we did not include habitat type while estimating the AWS. As far as your concern is on the application of Froude number for habitat classification, habitat type was only included in the modeling process while identifying significant linear and interactive terms that define the AWS. Thus, we adopted an accepted approach while estimating AWS that includes only velocity and depth parameters. We believe that our modeling and quantification are explicitly defined and systematically conducted. Thus, we believe that our approach adopts simple mathematical steps that are justifiable and acceptable in the flow management process. Methodology with this clarification is now updated in lines (206-212).

Reviewer #2: The recommendations suggested in the first stage of revision have been incorporated into the manuscript. Besides, it is an important contribution to the knowledge of threats to river dolphins in the Asian continent.

Thank you!
